# “COVID-19 and income inequality in OECD countries:” A methodological comment

**DOI:** 10.1007/s10198-023-01609-3

**Published:** 2023-06-23

**Authors:** Oded Stark

**Affiliations:** 1 University of Bonn, Bonn, Germany; 2 University of Warsaw, Warsaw, Poland

**Keywords:** Income inequality, Gini coefficient, Social stress, Forming public health policy, D01, D63, D91, I10, I14, I31, Z18

## Abstract

Wildman (2021), who identifies “a clear association between income inequality [measured by the Gini coefficient] and COVID-19 cases and deaths,” concludes that “a goal of government should be to reduce [income] inequalities and [thereby] improve [the COVID-19 outcomes /] underlying health of their populations.” In this Comment, we argue that reducing the Gini coefficient of the income distribution of a population need not weaken the population’s social stress. It is this stress which is a source of adverse health outcomes of the population. Because a measure of this stress is a *component* of the Gini coefficient, reducing the coefficient can leave the measure as is, or even increase the measure.

In a paper published in this journal, Wildman (2021), who identifies “a clear association between income inequality [measured by the Gini coefficient] and COVID-19 cases and deaths,” concludes that “a goal of government should be to reduce [income] inequalities and [thereby] improve [the COVID-19 outcomes /] underlying health of their populations.” The purpose of this Comment is to draw attention to a conceptual difficulty in this line of reasoning.

An association cannot serve as a basis for policy formation. An association between variable A and variable C could come about because variable B, which is part of variable A, is the cause of variable C. In such a circumstance, manipulation of variable C aimed at weakening the association between variables A and C - even if it shrinks variable A - may do that, while at the same time exacerbating variable B. The result will be exactly the opposite of the result aimed at when interference was contemplated.

We next translate this general observation to a specific statement, which we then follow with a numerical example. Without loss of generality, to begin with, we resort to an income distribution of a population of two persons.

Let $y = (y_{1},y_{2})$ be the vector of incomes of persons 1 and 2, respectively, where $y_{2} > y_{1}$. The Gini coefficient, $G$, of this income distribution is

$$ G = \dfrac{\dfrac{1}{2}(y_{2} - y_{1})}{y_{1} + y_{2}}. $$

The numerator here is a measure of the social stress that individual 1 experiences: it is the gap between the income of individual 2 and the income of individual 1, normalized by the size of this population. Because individual 2 does not experience social stress, the numerator is also the population’s social stress. The denominator is the population’s aggregate income. We use the notation $\mathit{AS}$ for aggregate (social) stress, and the notation $\mathit{AI}$ for aggregate income, and we apply subscripts 1 and 2 to indicate initial values and subsequent values, respectively. Thus,

$$ G_{1} = \dfrac{\mathit{AS}_{1}}{\mathit{AI}_{1}}. $$

Imagine now that aggregate income becomes $\mathit{AI}_{2}$, and that aggregate stress becomes $\mathit{AS}_{2} > \mathit{AS}_{1}$. It is straightforward to show that if

$$ \dfrac{\mathit{AS}_{2}}{\mathit{AS}_{1}} < \dfrac{\mathit{AI}_{2}}{\mathit{AI}_{1}} $$

then, as a result of these changes, the Gini coefficient decreases. The reason is that the preceding inequality is equivalent to the inequality

$$ \dfrac{\mathit{AS}_{2}}{\mathit{AI}_{2}} < \dfrac{\mathit{AS}_{1}}{\mathit{AI}_{1}}, $$

that is, to the inequality $G_{2} < G_{1}$. A decrease of the Gini coefficient can coincide with an increase of aggregate stress.

In order to see this happening, with the help of a numerical example, we let $y_{1} = 2$ and $y_{2} = 5$. Then $G_{1} = \dfrac{\dfrac{1}{2}(5 - 2)}{5 + 2}$. Suppose now that the two incomes $y_{1} = 2$ and $y_{2} = 5$ increase, respectively, to $\tilde{y}_{1} = 4$ and $\tilde{y}_{2} = 8$, in which case $G_{2}$, the revised Gini coefficient, is $G_{2} = \dfrac{\dfrac{1}{2}(8 - 4)}{8 + 4}$. By rewriting, $G_{1} = \dfrac{3}{14} = \dfrac{9}{42}$ and $G_{2} = \dfrac{4}{24} = \dfrac{7}{42}$, we see that while the Gini coefficient is lower, aggregate stress is higher: $\dfrac{1}{2}(8 - 4) = 2 > \dfrac{1}{2}(5 - 2) = \dfrac{3}{2}$.

If the announced aim of a policy of reducing income inequality by means of decreasing the Gini coefficient is to weaken a source of social stress, then, as just shown, the aim may not be achieved.

The preceding numerical illustration is even more striking than was already noted. This is because the lowering of the Gini coefficient is achieved while all incomes are raised. Thus, getting richer does not confer immunity against exposure to higher social stress.

When a population consists of three or more persons $n = 3,4,...$, with an income vector $y = (y_{1},...,y_{n})$, and where the incomes are ordered $0 < y_{1} < y_{2} < ... < y_{n}$, then the formula of the aggregate stress of a person whose income is $y_{i}$, $i = 1,2,...,n - 1$, is

$$ \mathit{AS}_{i} = \dfrac{1}{n}\sum _{j = i + 1}^{n} (y_{j} - y_{i}); $$

the social stress experienced by individual $i$ is expressed as the aggregate (sum) of the income excesses to which this individual is subjected. The individual at the lower end of the income hierarchy experiences the greatest social stress whereas the individual at the top of the income hierarchy experiences no social stress.

Multiplying and dividing the preceding expression by $n - i$, that is, rewriting $\mathit{AS}_{i}$ as

$$ \mathit{AS}_{i} = \dfrac{n - i}{n}\sum _{j = i + 1}^{n} \dfrac{y_{j} - y_{i}}{n - i}, $$

enables us to represent $\mathit{AS}_{i}$ as the product of two terms: the fraction of the population of those whose incomes are higher, and the mean excess income. With this alternative representation, there is yet another simple way of showing why a lower inequality of a population’s income distribution can coincide with elevated social stress.

Suppose that when the vector of incomes is ($2,3,4$), person 2 leaves the population. Income inequality goes down. However, the weight of income 4 in the income distribution increases: the fraction of the population of those whose income is higher - in this case this is the person whose income is 4 - increases. Person 3 senses higher social stress.

And if these examples are not striking enough, then there is a different way of presenting a configuration where a reduction of the Gini coefficient may not even dent $\mathit{AS}_{i}$, so that the policy prescribed by Wildman need not bite. Consider two income distributions: ($1,4$) and ($2,5$). Although there is no difference between the values of the numerator of the Gini coefficient in these two cases (in both cases $\mathit{AS}_{i} = \dfrac{3}{2}$), the Gini coefficient itself is smaller for ($2,5$) than for ($1,4$): $\dfrac{3}{14} < \dfrac{3}{10}$.

Perhaps one reason for Wildman (2021) focusing on income inequality as measured by the Gini coefficient as the target of policy intervention aimed at improving public health outcomes is that he considers the Gini coefficient to be the sum over $i = 1,2,...,n - 1$ of the levels of $\mathit{AS}_{i}$. Support for this conjecture is obtained from the following extract from a paper co-authored by Wildman. In Jones and Wildman (2008, p. 313) we read: “Summing the [$\mathit{AS}_{i}$] scores of each individual gives the Gini coefficient.” This is not so. Aggregating the levels of $\mathit{AS}_{i}$ of the members of a population does not “[give] the Gini coefficient;” rather, the aggregation gives *a component* of the Gini coefficient, namely the numerator of the Gini coefficient

$$ G = \dfrac{\displaystyle\sum_{i = 1}^{n - 1} \mathit{AS}_{i}}{\mathit{AI}}. $$

This distinction is crucial because, as we have shown, $\displaystyle\sum _{i = 1}^{n - 1} \mathit{AS}_{i}$ and $G$ need not move in the same direction.

In sum: steps taken to lower the Gini coefficient can actually exacerbate social stress, which is a cause of physical and mental harm. If the aim of public policy is to improve public health, then lowering the Gini coefficient may not be the right course of action. Wildman’s remarks that poverty and low income contribute to poor health outcomes do make sense, but a lowering of the Gini coefficient may not be an appropriate policy response to these conditions. As noted above, even if by raising incomes the Gini coefficient is reduced, social stress as a cause of adverse health outcomes may not be lowered.
